# In Silico Born Designed Anti-EGFR Aptamer Gol1 Has Anti-Proliferative Potential for Patient Glioblastoma Cells

**DOI:** 10.3390/ijms26031072

**Published:** 2025-01-26

**Authors:** Andrey Golovin, Fatima Dzarieva, Ksenia Rubetskaya, Dzhirgala Shamadykova, Dmitry Usachev, Galina Pavlova, Alexey Kopylov

**Affiliations:** 1 Belozersky Research Institute of Physical Chemical Biology, Lomonosov Moscow State University, 119991 Moscow, Russia; golovin.andrey@gmail.com; 2 Faculty of Bioengineering and Bioinformatics, Lomonosov Moscow State University, GSP-1, Leninskiye Gory, 1-73, 119234 Moscow, Russia; 3 Institute of Higher Nervous Activity and Neurophysiology, Russian Academy of Sciences, 117485 Moscow, Russia; ksusha21012002@yandex.ru (K.R.); djirgala04@gmail.com (D.S.); lkorochkin@mail.ru (G.P.); 4 Institution N. N. Burdenko National Medical Research Center of Neurosurgery of the Ministry of Health of the Russian Federation, 125047 Moscow, Russia; dousachev@nsi.ru

**Keywords:** brain tumors, glioblastoma, EGFR, EGFRvIII, aptamers, reverse folding task, molecular dynamics simulation, secondary structure predictions: transcriptomics

## Abstract

The epidermal growth factor receptor (EGFR) is one of the key oncomarkers in glioblastoma (GB) biomedical research. High levels of EGFR expression and mutations have been found in many GB patients, making the EGFR an attractive target for therapeutic treatment. The EGFRvIII mutant is the most studied, it is not found in normal cells and is positively associated with tumor cell aggressiveness and poor patient prognosis, not to mention there is a possibility of it being a tumor stem cell marker. Some anti-EGFR DNA aptamers have already been selected, including the aptamer U2. The goal of this study was to construct a more stable derivative of the aptamer U2, while not ruining its functional potential toward cell cultures from GB patients. A multiloop motif in a putative secondary structure of the aptamer U2 was taken as a key feature to design a novel minimal aptamer, Gol1, using molecular dynamics simulations for predicted 3D models. It turned out that the aptamer Gol1 has a similar putative secondary structure, with G-C base pairs providing its stability. The anti-proliferative activities of the aptamer Gol1 were assessed using patient-derived GB continuous cell cultures, G01 and BU881, with different abundances of EGFR and EGFRvIII. The transcriptome data for the cell culture G01, after aptamer Gol1 treatment, revealed significant changes in gene expression; it induced the transcription of genes associated with neurogenesis and cell differentiation, and it decreased the transcription of genes mediating key nuclear processes. There were significant changes in the gene transcription of key pro-oncogenic signaling pathways mediated by the EGFR. Therefore, the aptamer Gol1 could potentially be an efficient molecule for translation into biomedicine, in order to develop targeted therapy for GB patients.

## 1. Introduction

Glioma is the most common and aggressive type of brain tumor in adults [1]. The most aggressive form is glioblastoma (GB), a grade IV glioma, according to the WHO classification. GB is a highly malignant tumor characterized by rapid growth, extensive invasion, and poor prognosis. According to the WHO 2021 classification, GBs include adult diffuse astrocytic gliomas with wild-type IDH1/2. The diagnostic criteria for GB is based on the presence of one or more of three genetic parameters: (a) TERT promoter mutations, (b) EGFR gene amplification, and (c) copy number alterations +7/−10 [2]. GB accounts for approximately 15% of all brain tumors and 48.3% of all malignant tumors [3]. The 5-year survival rate of patients diagnosed with GB is 6.8% [3].

The incidence is approximately 3–4 cases per 100,000 people in the U.S. population per year [3]. GB is more common in the elderly, with a median age at diagnosis of 65 years [3]. There is a slight male predominance, with a male-to-female ratio of 1.6:1 [4].

Magnetic resonance imaging (MRI) is the primary imaging modality for the diagnosis and evaluation of GB [5]. GBs, visualized by MRI, show a heterogeneous contrast-enhancing mass, with central necrosis and peripheral edema. A definitive diagnosis of GBs is made based on histological analysis of a tissue biopsy or tumor resection, as well as molecular genetic analysis.

The standard of care first diagnosed is a maximal safe surgical resection, followed by chemoradiotherapy combined with temozolomide and then adjuvant temozolomide therapy [6]. This standard has not changed significantly over the past 20 years, although it is ineffective and has only increased the median patient survival by approximately 2.5 months [6,7].

Unfortunately, frequent recurrences are observed after surgical tumor resection in GB patients [8,9]. The high recurrence rate may be due to the presence of unresectable foci of residual tumor cells removed from the neoplasm early in the formation of the tumor [10] or the invasion of tumor cells into healthy brain tissue [11].

Other therapies, such as tumor treating fields (TTFs) [12,13,14], immunotherapy [15,16], and targeted molecular therapy [17], have been actively studied in clinical trials, but have yet to demonstrate a meaningful effect on highly malignant gliomas which alters the course of the disease.

Despite aggressive treatment, the prognosis for GB remains poor, with a median overall survival of 14–16 months after diagnosis [18]. Overall, glioblastoma is an extremely aggressive brain tumor, with a poor prognosis. Ongoing research is aimed at better understanding the biology of the disease and developing more effective treatment strategies.

Recently, aptamer-based research has become widespread for more effective theranostics (a personalized medicine approach that combines diagnosis and therapy) for various tumor types. Aptamers are short single-chain nucleic acids that can bind with high specificity and affinity to various targets, such as proteins, small molecules, and cellular components, etc. [19,20,21]. Their unique properties make them a powerful tool for the diagnosis and treatment of various tumors, including gliomas.

Aptamers can be specifically designed for tumor-targeted therapy, allowing drugs to be precisely directed at tumor cells, thus minimizing their effects on healthy tissues [19]. Importantly, aptamers have the unique ability to distinguish between similar targets, such as isomers of chemical compounds or point mutations manifested in protein molecules [22,23]. This is particularly important in the treatment of aggressive forms of cancer, where maximum treatment efficacy with minimal side effects is required.

It is also worth noting that aptamers can be easily modified to improve their properties, such as their stability, affinity, and specificity, making them a convenient tool for personalized tumor therapy [24,25,26]. With the ability to rapidly synthesize and modify aptamers, researchers and clinicians have the ability to tailor aptamers to specific tumor types and patient characteristics.

In tumor diagnostics, aptamers can be used to detect tumor markers and biomarkers, allowing for more accurate and sensitive patient screening [27,28,29]. Specific binding of aptamers to tumor cells or proteins can also be used to form contrast agents in radiology or as labeled particles in imaging techniques [30].

Aptamers can be used as drug carriers for tumor treatment, ensuring their delivery to the tumor with high precision and minimizing side effects on healthy tissues [31,32]. Moreover, aptamers can also act as inhibitors of signaling pathways, helping to suppress tumor growth or induce the apoptosis of tumor cells [32,33].

Rapid progress and advances have been made in the use of aptamers in the diagnosis and treatment of tumors. Technologically innovative, this technology provides great promise for developing pioneering ways to fight cancer, including glioblastoma. In the future, aptamers may become a key component of a personalized approach to cancer treatment, enabling precise and effective targeting of tumor cells with a minimal impact on healthy tissue.

EGFR is one of the markers of glioblastoma and may be an effective target protein for aptamers. The EGFRvIII mutation is of particular interest to research as it correlates with a poor prognosis for patients. In 2014, Wu et al. [34] selected DNA aptamers against U87-EGFRvIII cells, i.e., specific for the EGFRvIII receptor, using the whole-cell systematic evolution of ligands by exponential enrichment (SELEX) method. The U2 and U8 aptamers were shown to have the best binding capacity to the EGFRvIII receptor protein.

EGFR is a receptor tyrosine kinase that plays a key role in the regulation of cell proliferation, survival, and differentiation [35,36]. In GB, genetic alterations such as amplification, mutation, and overexpression of the EGFR gene lead to hyperactivation of the signaling pathway, promoting tumor growth and progression. The most common genetic alteration observed in GB involving EGFR is a mutant form known as EGFRvIII [37].

EGFRvIII is a constitutively active truncated form of the EGFR receptor characterized by a deletion of exons 2 to 7 that lacks a ligand binding domain [38]. This mutation is associated with persistent activation of the EGFR signaling pathway, resulting in uncontrolled cell proliferation and tumor growth. Studies have shown that EGFRvIII expression is associated with increased tumor aggressiveness, resistance to therapy, and poor prognosis in patients with GB [38]. Several studies classify EGFRvIII as a potential cancer stem cell marker [39]. This form of the receptor is not found in normal cells [40].

In 2018, Zhang et al. [41] showed that the U2 aptamer can specifically bind to U87-EGFRvIII cells and internalize into the cells through the endosome recycling pathway using FCM and immunofluorescence techniques. The effect of the U2 aptamer on U87MG/EGFRvIII cells was also demonstrated: 24 h treatment of U87-EGFRvIII cells with the U2 aptamer significantly reduced tumor cell proliferation, migration, and invasion and the activation of apoptosis. However, U87MG/EGFRvIII cells treated with U2 at concentrations of 25 and 50 nM showed only a 59% and 51% reduction in tumor cell viability, respectively. Likely, the survival of 41% to 49% of tumor cells is due to the large size of the aptamer and, correspondingly, poor penetration into tumor cells.

In this work, we present a modification of the U2 aptamer, Gol1. The shortened Gol1 aptamer was derived from the secondary structure of the U2 aptamer as a result of sequence optimization. Secondary structure elements of U2 were preserved in Go11 which allowed Go11 to retain its high specificity for targeting the EGFR and EGFRvIII receptors. This aptamer might be a promising molecule for further therapies and imaging techniques for glioblastoma.

## 2. Results

### 2.1. Computational Modeling and Synthesis of the Gol1 Aptamer

**Figure 1:**
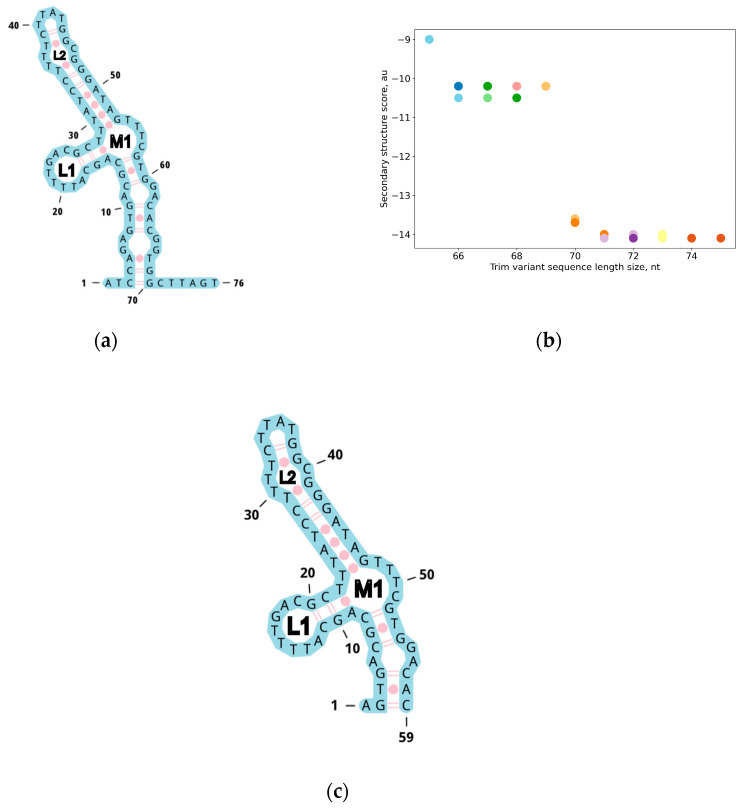
(**a**) Secondary structure of the U2 aptamer; (**b**) Change in formation of the secondary structure energy when the aptamer is shortened. Data are shown for structures where the multiloop is preserved; (**c**) Truncated secondary structure of the U2 aptamer.

The cell SELEX method (Cell-SELEX Aptamer for Highly Specific Radionuclide Molecular Imaging of Glioblastoma In Vivo) was used to search for DNA oligonucleotides that could bind EGFR and EGFRvIII on the cell surface of the U87MG cell line [41]. The four aptamers U2, U8, U19, and U31 bind to EGFRvIII in the nanomolar range, making these sequences attractive for the further development of EGFR inhibitors [41]. The sequences of these aptamers contain poly-T repeats, which are expected to negatively affect the formation of stable secondary structure elements. The goal of this work was to minimize the sequence while preserving the secondary structure elements. Based on a comparison of the putative secondary structures of the listed aptamers, the aptamer U2 was of particular interest because its putative secondary structure contains a “multiloop” element, while the other aptamers form hairpins with internal loops (Figure 1a).

We used Python bindings for the ViennaRNA package [42] to resolve the problems of length minimization and secondary structure stabilization. We used the DNA_Mathews2004 parameter set [43] for the parameterization of the DNA secondary structure score. To achieve reproducibility, all activities were recorded using Jupyter Notebook [44] Positioning and Power in Academic Publishing: Players, Agents, and Agendas (pp. 87–90). We proposed considering loops 1 and 2 and a multiloop in the 50–60 nucleotide range in U2 as putative recognition elements.

A dot-bracket notation description of the multiloop was chosen as the key pattern for the selected structure. Using a sliding window, we searched for variants of sequence truncation while preserving the multiloop substructure (Figure 1b). This resulted in a shortening of 6 nucleotides from the 5′ end and 11 nucleotides from the 3′ end (Figure 1c). This result demonstrates that shortening a sequence while preserving its predicted secondary structure “on paper” can lead to inherently false results, i.e., the formation of an entirely different structure.

The minimal sequence and its secondary structure containing loops 1 and 2 and the M1 multiloop were used to search for sequences that could form the desired secondary structure [45]. Formally, the diversity of sequences that could reproduce the minimal structure can be estimated as 4 raised to the power of the number of nucleotide pairs in the stem regions, 415 or about 10^9^. It is evident that replacing A-T pairs with G-C pairs should yield the desired result, but one must avoid the formation of quadruplex motifs and pseudoknots, which is also difficult to accomplish “on paper”. The chosen approach has a stochastic element, and, therefore, we repeated the procedure 1000 times to achieve sample saturation. The results yielded 522 unique sequences.

**Figure 2:**
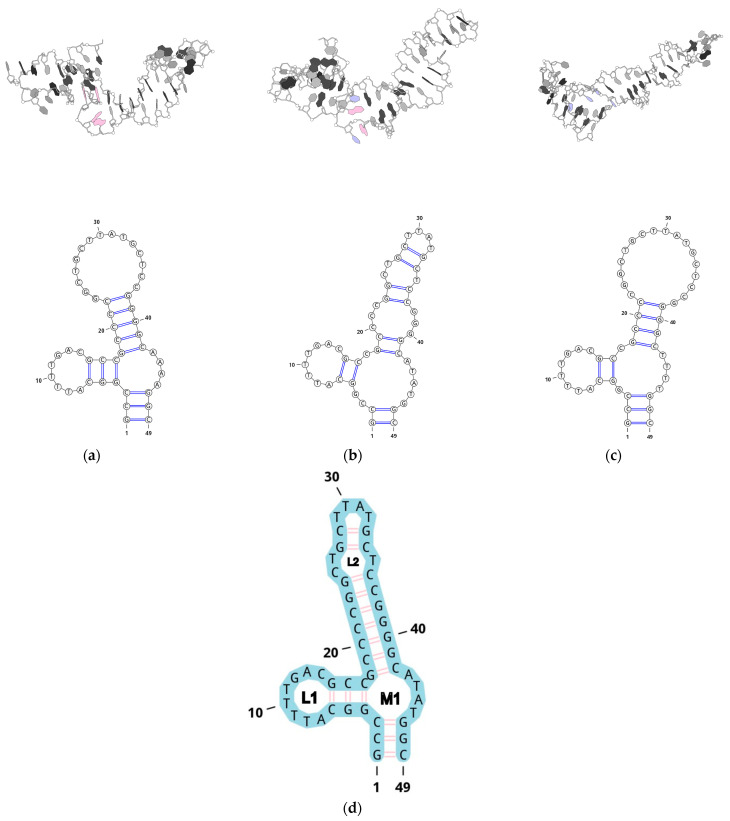
Optimized 3D models of the aptamer with different multiloop compositions: (**a**) AAAA, (**b**) ATAT, and (**c**) TTTT: (**d**) Secondary structure of the Gol1 aptamer. The pink labeled nucleotides are adenine (A) and the blue ones in the loop are thymine (T). The aptamer model with the AAAA multiloop sequence (Figure 2a) forms a sufficiently large surface area at the base of the stack for potential interaction with the EGFR protein, which upon dimerization forms a distinct plane with a surface area containing a high density of positive charge (Figure S1). All purine bases form stacking interactions within the loop and its surroundings. This leads to a situation where only the sugar-phosphate backbone of the loop can interact with the protein, resulting in the loss of interactions involving heterocyclic bases, which could contribute significantly to the aptamer’s specificity. The model aptamer with the ATAT sequence forms a similar surface but with the adenine and thymine bases 45–46 facing potential interactions with the protein, which is attractive for the formation of additional stacking interactions and/or hydrogen bonds. In the case of the TTTT sequence, the multiloop structure becomes unstable and the aptamer is unable to form an interaction plane.

For each design result, a truncated design variant was searched. After sorting, the best design variant was edited to recover the original sequences in loop 1 and loop 2. Since most of the designs had adenine residues in the multiloop, it was decided to build 3D models with different compositions of the multiloop: AAAA, TTTT, and ATAT. The models were constructed using trRosettaRNA as the RNA structure, the resulting structures were modified to match the DNA and equilibrated using metadynamics by the collective variable ERMSD for the loop bases. The choice of this sequence of actions is dictated by the extremely low amount of information about complex DNA structures in the PDB database, which makes it impossible to apply machine-learning models to construct the DNA structure directly. We tried to use AlpaFold 3 for this purpose and the result for the aptamer structure was unsatisfactory: the pIDDT score was less than 50 for all DNA residues. It has been previously shown that by using modern force fields, the transition from the A-form of DNA to the B-form is quite fast in molecular dynamics [46]. We performed molecular dynamics simulations in five iterations with a trajectory length of 1 µs for three candidate sequences. The consensus relaxation results of the models in an explicitly defined solvent are shown in Figure 2. The final secondary structure of the Gol1 aptamer is shown in Figure 2d.

The variant with the ATAT sequence in the loop exhibits higher conformational mobility (Figure S1). When analyzing the RMSD (root mean square deviation of atomic positions after structure fit) change from the 20 ns backward trajectory state, the AAAA and TTTT variants exhibit RMSD values below 4A after 250 ns of simulation, while in the case of ATAT RMSD fluctuations are observed at 6A.

Therefore, we selected the ATAT sequence for the multiloop, and the final sequence of the whole aptamer was named Gol1. The estimated secondary structure formation energy of GOL1 was −24.7 AU, while the best design had −26.1 AU. The secondary structure formation energy of the original U2 was −14.1 AU.

### 2.2. Human Glioblastoma Cell Cultures with EGFRwt/EGFRvIII Expression

**Figure 3:**
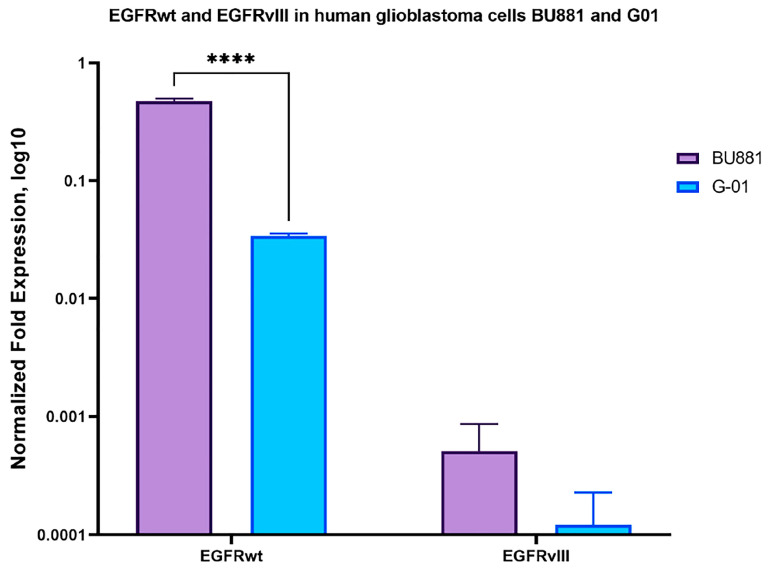
Expression levels of EGFRwt and EGFRvIII in BU881 and G01 human glioblastoma cell cultures by RT-qPCR. Data are presented as mean ± SD; n = 3 for each group. A statistically significant differences between groups are indicated by asterisks (multiple unpaired *t*-tests, **** = *p* < 0.0001).

Two human glioblastoma cell cultures, BU881 and G01, derived from patient tumor tissue, were used to evaluate the effect of aptamers on human glioblastoma cell proliferation. Both cell cultures show high expression levels of EGFRwt, as determined by RT-qPCR (Figure 3).

qPCR data showed that the expression of EGFRwt in the human glioblastoma cell culture BU881 is significantly higher than in G01 cells. There is also a three-fold increase in EGFRvIII expression in BU881 when compared to G01 cells (Figure 3).

### 2.3. Analysis of the Effect of Aptamers on the Proliferative Potential of Human Glioblastoma Cells

**Figure 4:**
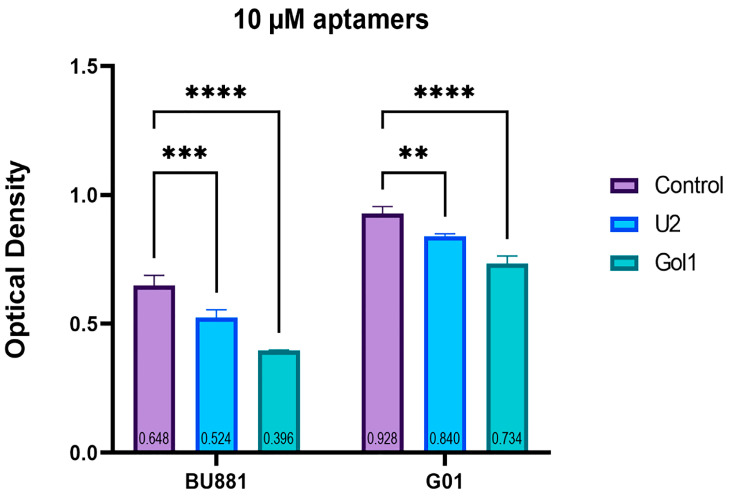
Evaluation of proliferation change in human glioblastoma BU881 and G01 culture cells treated with the aptamers U2 and Gol1 at a concentration of 10 μM by MTS assay. Statistically significant differences between control and experimental groups are indicated by asterisks (one-way analysis of variance (ANOVA) followed by Bonferroni multiple comparison test, ** = *p* < 0.01, *** = *p* < 0.001, **** = *p* < 0.0001).

To evaluate the effect of the U2 and Gol1 aptamers on the proliferative potential of cells with variable EGFRwt and EGFRvIII receptor gene expression, BU881 and G01 cells were analyzed by MTS assay (Figure 4).

Interestingly, it was noted that the greater the representation of target receptors is in the cell culture, the more pronounced the antiproliferative effect of both aptamers (Figure 4). Moreover, in both cultures, the Gol1 aptamer reduced the proliferation of human glioblastoma cells more effectively compared to the U2 aptamer. In G01 cell culture, the aptamer U2 at a concentration of 10 μM reduced cell proliferation by ~10% and the aptamer Gol1 by ~21%; correspondingly, in human glioblastoma BU881 cell culture cells at the same concentration, the aptamer U2 reduced proliferation by ~20% and the aptamer Gol1 by ~40%. Thus, in the human glioblastoma BU881 cell culture with a more pronounced expression of target receptor genes, the antiproliferative effect of both experimental aptamers is two-fold more pronounced when compared to Gol1 cells. In both cell cultures, the Gol1 aptamer is two-fold more effective than the U2 aptamer.

**Figure 5:**
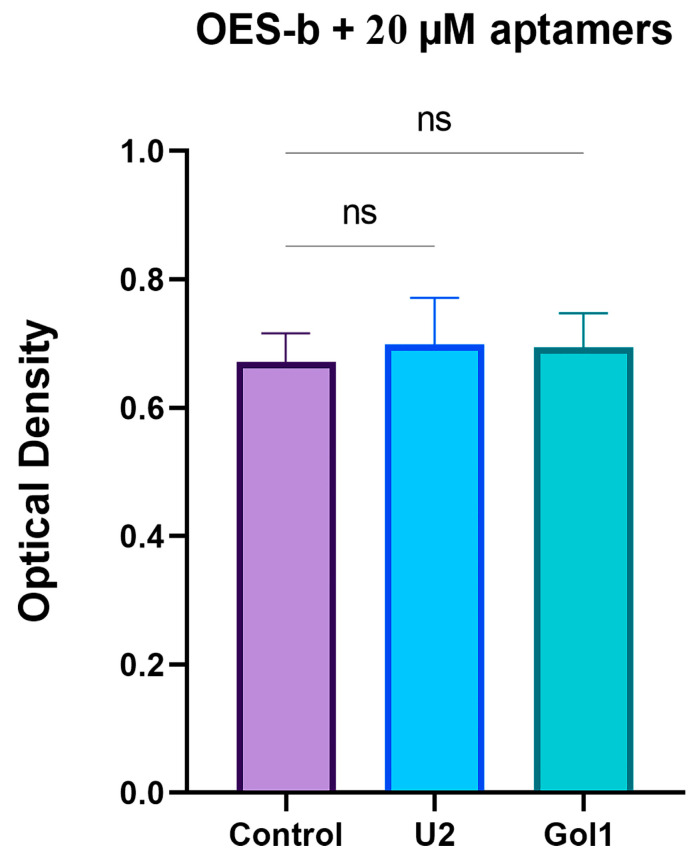
Evaluation of proliferation changes in OES-b treated with U2 and Gol1 aptamers at a concentration of 20 μM by MTS assay. A statistically significant differences between control and experimental groups were evaluated by one-way analysis of variance (ANOVA) followed by Bonferroni multiple comparison test (ns—no statistically significant differences).

To assess whether the antiproliferative effect of aptamers is specific to tumor cells, we investigated the effect of U2 and Gol on the proliferative potential of OES-b olfactory neuroepithelial lining cells (Figure S2) by MTS test (Figure 5).

OES-b cells were treated with the aptamers at a concentration two times higher than that used in the human glioblastoma cell experiments (Figure 5). However, even at the higher concentration, neither aptamer had a statistically significant effect on the level of cell proliferation compared to the control.

These results suggest that the aptamers specifically target the EGFRwt and EGFRvIII receptors of tumor cells. The aptamer Gol1 has a more pronounced antiproliferative effect on human glioblastoma cells compared to the aptamer U2, while both aptamers do not affect normal cells.

### 2.4. Analysis of Transcriptomic Data of Human Glioblastoma G01 Cells After Gol1 Aptamer Treatment

**Figure 6:**
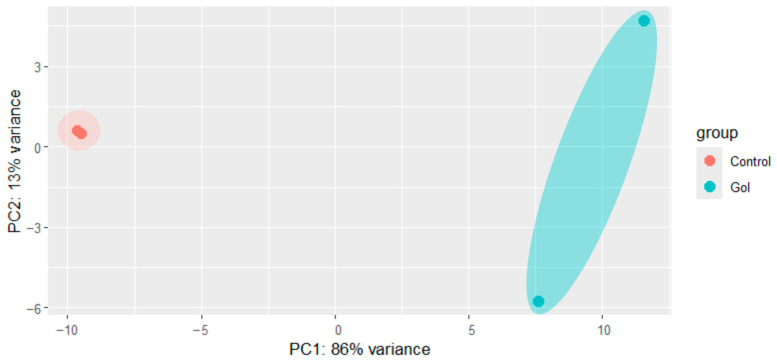
PCA plot in control and experimental groups consisting of two transcriptome analysis datasets of human glioblastoma G01 cells with and without Gol1 aptamer treatment.

The effect of the Gol1 aptamer on the transcriptome of human glioblastoma tumor cells, G01, was evaluated. Transcriptome analysis of human glioblastoma cells, G01, was performed after incubation with the Gol1 aptamer at a concentration of 10 μM for 72 h. Based on the transcriptome results, incubation with the Gol1 aptamer resulted in profound changes in gene expression, effectively separating treated and untreated human glioblastoma G01 cells as observed in the Principal Component Analysis (PCA) plot (Figure 6).

**Figure 7:**
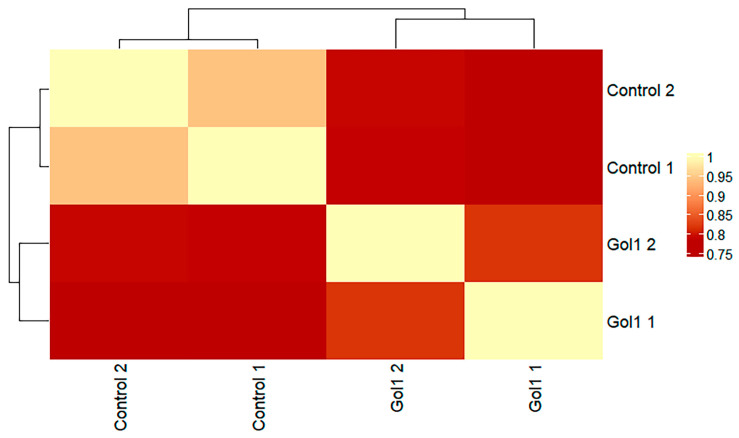
Heat map analysis summarizing the transcriptional changes in human glioblastoma G01 cells with and without Gol1 aptamer treatment.

**Figure 8:**
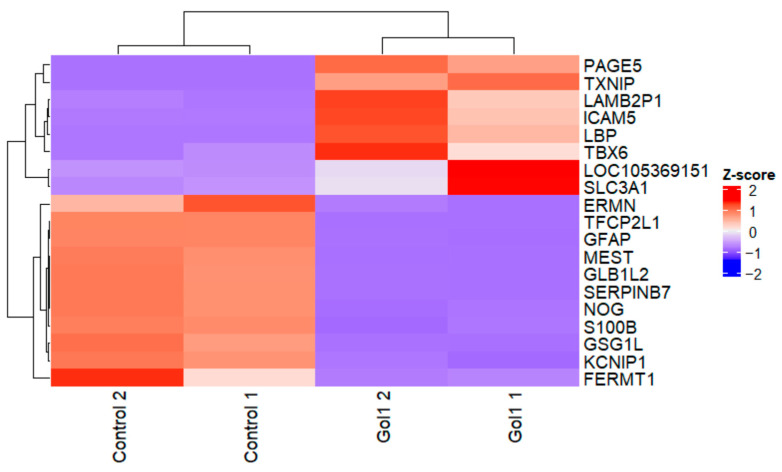
Heat map analysis summarizing transcriptional changes in human glioblastoma G01 cells with and without Gol1 aptamer treatment for genes with a log2FC modulo value greater than 4. Gene expression was log2 transformed and shown as a normalized z-score.

Next, we focused on detecting differentially expressed transcripts of Gol1 aptamer-treated and untreated human glioblastoma G01 cells. Heat map analysis effectively separated treated and untreated human glioblastoma G01 cells (Figure 7 and Figure 8), where a clear clustering can be observed confirming the effect of the Gol1 aptamer on gene expression of human glioblastoma G01 cells (Figure 6).

**Figure 9:**
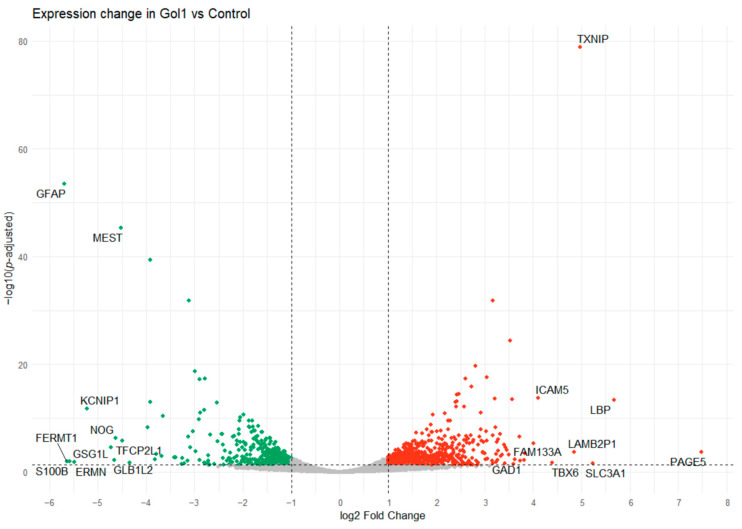
Volcano plot analysis showing statistically significant differentially expressed (DE) genes after treatment of human glioblastoma G01 cells with the Gol1 aptamer. The y-axis represents the *p*-value after log10 transformation, and the x-axis represents the multiple transformed to log2. Horizontal line: −log10(0.05) Vertical line: log2FoldChange value (log2 ratio of gene expression levels in the compared lines) equal to −1 and 1.

A total of 50,006 genes were analyzed. Next, only the genes with an average number of reads across samples greater than 10 were selected. This resulted in the selection of 12392 genes which represent ~25% of the original number of genes. Volcano plot analysis identified 1207 statistically significant differentially expressed (DE) genes in human glioblastoma G01 cells (Figure 9).

After DE analysis, genes with adjusted significance levels *p* < 0.05 and |log2FoldChange| > 1 were selected. In total, 765 genes were upregulated and 442 genes were downregulated in the sample treated with Gol1, representing 63.3% and 36.7% of the total DE genes, respectively (Figure 9).

**Figure 10:**
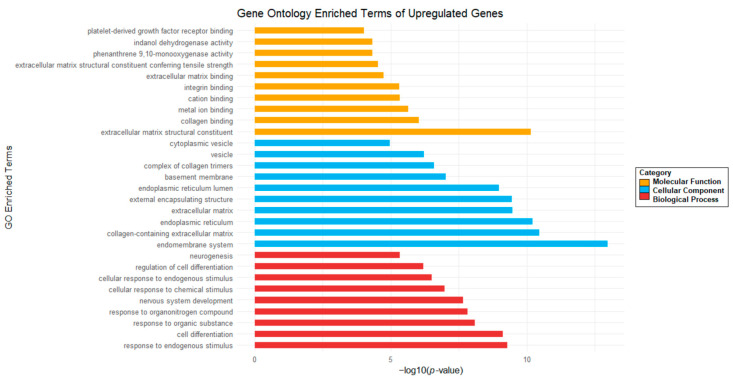
Gene Ontology (GO) analysis of upregulated genes in human glioblastoma G01 cells after Gol1 aptamer treatment.

To correlate the observed transcriptional changes in human glioblastoma G01 cells after Gol1 aptamer addition with biological processes, we performed Gene Ontology (GO) analysis. GO analysis of DE transcripts of upregulated genes (Figure 10) shows an increase in the expression of genes responsible for neurogenesis and cell differentiation. In addition, there is an increase in pathways related to the cellular response to various stimuli (endogenous, chemical, and organic).

All statistically significant GO pathways of upregulated genes are shown. They belong to one of the following GO categories: Molecular Function (MF) in yellow bars, Cellular Components (CC) in blue bars, or Biological Processes (BP) in red bars.

**Figure 11:**
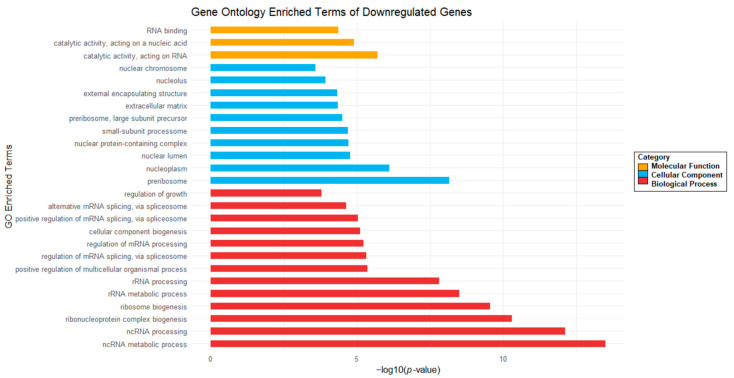
Gene Ontology (GO) analysis of downregulated genes in human glioblastoma G01 cells after Gol1 aptamer treatment.

In turn, the GO analysis of DE transcripts of downregulated genes (Figure 11) shows decreased expression of genes involved in cell growth pathways, RNA splicing, alternative RNA splicing, RNA processing, biogenesis of various nuclear components, and nuclear metabolic processes.

All statistically significant GO pathways of downregulated genes are shown. They belong to one of the following GO categories: Molecular Function (MF) in yellow bars, Cellular Components (CC) in blue bars, or Biological Processes (BP) in red bars.

Since the target molecule of the aptamers is EGFR, we analyzed the gene expression changes of the major signaling pathways mediated by the EGFR receptor. The major pro-oncogenic signaling pathways of the EGFR receptor include the MAPK pathway, PI3K-Akt pathway, and JAK-STAT pathway [47].

Out of 246 genes known to be involved in the regulation of the MAPK pathway, 23 DE genes are upregulated and 4 DE genes are downregulated. In the case of the PI3K-Akt pathway, 30 DE genes out of 338 genes known to be involved in the PI3K-Akt pathway are upregulated, and 13 genes are downregulated. Finally, in the gene cascade of the JAK-STAT pathway, 10 genes out of 162 genes are overexpressed and 6 genes of them are downregulated.

**Figure 12:**
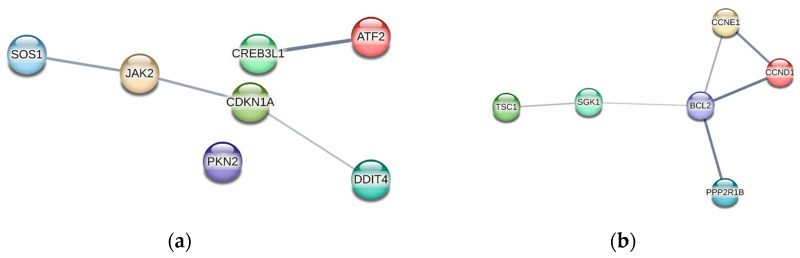
PPI network of PI3K-Akt cascade genes, (**a**) overexpressed and (**b**) hypo-expressed in the human glioblastoma G01 cell prototype after exposure to the Gol1 aptamer, obtained by STRING. PPI, protein–protein interaction; STRING, a search tool to extract interacting genes.

The most pronounced changes were observed in the genes of the PI3K-Akt pathway cascade (Figure 12).

## 3. Discussion

Aptamer-based studies for GB therapy are important for several reasons. First is the specificity of action: Aptamers can be designed to precisely recognize and bind to molecules that play a key role in the development of GB [23,48]. The use of aptamers allows the targeting of specific proteins or processes within tumor tissue, minimizing side effects on healthy tissue. Second is the personalized approach: Due to the specificity of aptamer action, it is possible to create personalized therapies that consider individual patient and tumor characteristics. This helps to improve treatment efficacy and reduce the risk of complications. Third is overcoming drug resistance: GB is often resistant to standard chemotherapeutic agents [49]. Aptamers are a new class of drugs that offer the possibility of overcoming drug resistance in the treatment of glioblastoma. Fourth is drug delivery: aptamers can be used to deliver drugs directly to GB tumor cells or tumor vessels by penetrating the blood–brain barrier (BBB), thereby increasing the efficacy of therapy and providing a more targeted effect on the tumor [48,50].

GB is characterized by the overexpression and dysregulation of the EGFR signaling pathway [51]. A mutant form of the EGF receptor, EGFRvIII, is found in 60% of GB cases [37]. EGFRvIII is positively associated with tumor malignancy and aggressivity [52] and is one of the potential markers of cancer stem cells [39]. What makes the mutant receptor an even more attractive target for GB therapy and diagnosis is that normal cells are not characterized by its expression [40].

Our previous studies have shown that in competitive apta-immunocytochemical staining of the patient glioblastoma cell culture G01, the aptamer Gol1 showed a higher competitive binding to EGFRvIII compared to the aptamer U2 and a commercial anti-EGFRvIII antibody, and in the case of EGFRwt it was equal to the antibody [53]. In this study, we proposed and synthesized an aptamer Gol1 to EGFR and EGFRvIII, which is a variant sequence capable of forming a secondary structure similar to the U2 aptamer known in the literature [41]. Using the MTS assay method, we evaluated changes in the proliferative potential of the human glioblastoma culture cells G01 and BU881, which are characterized by different expression levels of the two target receptors, EGFR and EGFRvIII. As expected, there is a correlation between the cell proliferation rate and the level of receptor expression (Figure 4). In addition, the Gol1 aptamer exhibits a more pronounced antiproliferative effect than the U2 aptamer in both cultures. These data demonstrate that the new variant of the Gol1 aptamer we have proposed is a more potent molecule compared to its predecessor.

Presumably, the Gol1 aptamer promotes the overexpression of several components of the PI3K-Akt pathway that are positively associated with apoptosis and differentiation and negatively associated with proliferation and cell growth in human glioblastoma cells, such as the CDKN1A and PKN2 genes (Figure 12).

The p21 protein, encoded by the CDKN1A gene, is a major member of the cyclin-dependent kinase (CDK) inhibitors and plays an important role in the cell cycle regulation that contributes to genomic stability [54]. This protein is frequently de-regulated in several human cancers [55,56]. In addition, p21 plays important roles in processes such as apoptosis, differentiation, reprogramming of induced pluripotent stem cells, DNA repair, transcription, and cell migration [55,56]. p21 is critical for the transition between the G2 and M phases of the cell cycle, and its deficiency can lead to the prolongation of mitosis, which, in turn, can cause mitotic dysfunction and consequent genomic instability [57]. p21 inhibits CDKs that phosphorylate the retinoblastoma protein (pRB)-related proteins p107 and p130. Thus, the expression of p21/CDKN1A results in the hypophosphorylation of p107 and p130. In this hypophosphorylated state, p107 and p130 can bind to other proteins to form the DREAM complex and thereby repress transcription. This, in turn, can lead to irreversible cell cycle arrest as a result of senescence or induction of apoptosis [58]. In addition, p21 has been shown to repress a relevant number of genes that control the S-phase and mitosis. Thus, the activity of p21 as an inhibitor of cell cycle progression would be mediated not only by CDK inhibition but also by the transcriptional regulation of key genes [59]. This protein can also directly inhibit cell proliferation by binding to CDKs [60] and proliferating cell nuclear antigen (PCNA) [61].

Protein kinase C-related kinase 2 (PKN2/PRK2) is involved in the regulation of various biological processes including cell migration, adhesion, and death. Cell proliferation and differentiation are mutually exclusive processes that are regulated by different extracellular signals that trigger the activation of often-identical intracellular signaling pathways such as Akt signaling [62]. PKN2 is thought to be specifically involved in differentiation-specific Akt activation and myoblast differentiation. PKN2 expression is increased during myoblast differentiation, whereas PKN2 overexpression did not show increased Akt activation in proliferating cells [63]. A protein–protein interaction between Akt and the C-terminal region of PKN2 specifically reduces Akt protein kinase activity, resulting in the inhibition of downstream Akt signaling in vivo. The C-terminal fragment of PKN2 strongly inhibits the Akt-mediated phosphorylation of BAD, a pro-apoptotic Bcl-2 family protein, and blocks the anti-apoptotic activity of Akt in vivo [64].

When we analyzed the genes of the PI3K-Akt pathway, whose expression is downregulated in human glioblastoma G01 cells after Gol1 aptamer treatment (Figure 12), we observed several very interesting trends. The expression of the key genes that are associated with high levels of malignancy and poor prognosis in GB (PP2R1B, CCND1, CCNE1, BCL2, and SGK1) was reduced. Furthermore, we identified BCL2 and SGK1 which are known to be the key anti-apoptotic genes [65,66,67], as well as CCND1 and SGK1 genes, the markers of human GB stem cells [68,69,70].

Protein phosphatase 2 scaffold subunit Abeta (PPP2R1B) is a gene encoding the beta isoform of serine/threonine protein phosphatase 2A subunit A (PP2A). Canonically, PP2A functions as a tumor suppressor gene; however, several studies have shown that the inhibition of PP2A activity has an anti-oncogenic effect. For example, PP2A inhibition leads to the increased radiosensitivity of tumor cells and the prevention of tumor recurrence. In malignant gliomas, PP2A inhibition increases the frequency of cells in M-phase mitosis, leading to an inhibition of tumor proliferation [71]. A key problem, however, is the selection of a specific PP2A inhibitor that does not affect normal cells. We have shown that the Gol1 aptamer does not affect normal cells, making it a valuable candidate for PP2A inhibition. In GB, genetic alterations in genes encoding PP2A subunits are rare (less than 1%) according to the Cancer Genome Atlas (TCGA) datasets [37]. Non-genetic mechanisms of PP2A dysregulation in GB might be due to the overactivation of receptor tyrosine kinases (RTKs) such as EGFR [72].

Cyclin D1 and cyclin E1 are regulators of G1–S-phase cell cycle progression, are often constitutively expressed, and are associated with pathogenesis and tumorigenesis in most human cancers. They are considered to be promising targets for cancer therapy [73,74].

CCND1 is a gene encoding the protein cyclin D1, which is overexpressed in malignant gliomas and positively associated with malignancy grade and poor prognosis [75]. In a 2020 study, CCND1 expression was shown to be significantly upregulated in GB tissues and GB-derived stem cells [68]. The inhibition of CCND1 renders tumor cells more sensitive to temozolomide (TMZ) treatment and temozolomide-induced apoptosis [76].

CCNE1 is the gene encoding the protein cyclin E1, a nuclear protein required for cell cycle progression [77,78], DNA replication [79,80], and centrosome duplication [81]. The expression of this gene correlates with the malignancy rate of meningiomas [82]. The inhibition of cyclin E1 has been reported to significantly improve the efficacy of temozolomide therapy for GB [83].

The BCL-2 gene plays a key role in the regulation of apoptosis, encoding pro-apoptotic proteins (such as Bax and Bak) and anti-apoptotic proteins (such as BCL-2 itself). BCL-2 functions mainly as an inhibitor of apoptosis by preventing the escape of cytochrome c from the mitochondria into the cytosol [84]. BCL-2 is a prognostic factor for highly malignant gliomas [85]. One of the hallmarks of GB is the abnormal expression of anti-apoptotic proteins such as BCL-2, which is associated with tumor survival and the resistance of malignant cells to radiotherapy and chemotherapy [65,66]. The development and clinical trials of BCL-2 inhibitors such as Venetoclax have demonstrated efficacy in reducing cancer cell survival by restoring apoptosis [86]. Combining BCL-2 inhibitors with other therapies, such as chemotherapy or radiotherapy, may enhance their effects [66,87].

Serum and glucocorticoid-induced protein kinase 1 (SGK1) is a serine-threonine kinase involved in various cellular processes including cell survival, growth, and apoptosis [88,89,90]. SGK1 is activated by several growth factors and stress signals. It promotes cell survival by inhibiting apoptotic pathways [67,91,92]. For example, SGK1 can influence the balance between pro- and anti-apoptotic members of the BCL-2 family [93]. Increased SGK1 expression correlates with increased tumor cell proliferation, invasion, and resistance to apoptosis, contributing to the aggressive nature of tumor cells [67,69,93]. Studies have shown that SGK1 is frequently overexpressed in GB cells [91] and is a key gene for GB stem cell viability [69,70]. SGK1 may play a role in glioblastoma resistance to conventional therapies [91,94].

Of particular interest among MAPK pathway genes whose expression is altered upon treatment with the Gol1 aptamer is the overexpression of DUSP5 and DDIT3 and the decreased expression of RRAS2 genes.

Dual-specificity phosphatase 5 (DUSP5) plays an important role in cell proliferation and differentiation by negatively regulating members of the MAPK superfamily (MAPK/ERK, SAPK/JNK, and p38) [95]. Furthermore, DUSP5 acts as a negative regulator of glioma cell motility and the ERK signaling pathway [96]. DNA damage-inducible transcript 3 (DDIT3) is a pro-apoptotic transcription factor encoded by the DDIT3 gene. Increased DDIT3 expression has been shown to be associated with the activation of apoptosis in GB tumor cells [97]. Ras-related protein (RRAS2), encoded by the gene of the same name, is a member of the Ras family of small GTPases. The RRAS2 gene is overexpressed in many malignancies, including brain tumors and especially GB [98,99,100]. Recently, Gutierrez-Erlandsson S et al. demonstrated that RRAS2 is an important driver of neuronal transformation in tumorigenesis [100].

Our research findings provide an insight into the potentially important contribution that the Gol1 aptamer can make to the diagnosis and therapy of glioblastoma. We believe this approach may become the key to resolving currently acute problems in glioblastoma diagnostics and treatment based on the ability of aptamers to target specific molecules without normal tissue damage. We consider this to be a very promising approach in tumor theranostics.

## 4. Materials and Methods

### 4.1. Aptamers

To find a new DNA sequence, we decided to optimize the secondary structure of known aptamers. As a source of secondary structure data, we used the predicted states for the DNA aptamer U2 selected by the team of X. Zhang [34]. Based on the analysis of predicted structures of two different aptamers, U2 and U31, specific for EGFR and EGFRvIII, the design of a new structure containing a multiloop was selected. It was assumed that the multiloop serves as the recognition element. Based on the results of the computational optimization of the sequence for the desired secondary structure by multidimensional Boltzmann scanning in the RNASketch utility [101,102], the Gol1 aptamer was proposed with the sequence: 5′-GCCGGCGGCATTTTGACGCCGCCGCCGGCCGGCTGCTTATGCTGCTCCGGGGGGCATATATGGC-3′.

### 4.2. Modeling

DNA secondary structure prediction was performed using the ViennaRNA package [42]. The DNA_Mathews2004 parameter set was used to parameterize the DNA secondary structure estimation. The visualization of the RNA secondary structure was performed using the Varna applet [103]. The inverse folding (design) procedure (inverse_pf_fold) from the ViennaRNA package was used. We used Python bindings for the ViennaRNA package to solve the length minimization and secondary structure stabilization problems. All steps were recorded in Jupyter Notebook to achieve reproducibility.

For 3D structure prediction, we used trRosettaRNA [104] with the input sequence and probable secondary structure; after 10 replicates, 3D structures preserving the original secondary structure were used for modeling.

MD simulations: We ran three sets of simulations for each “junction” content: AAAA, ATAT, and TTTT. Each system was run in three independent replicates of 1000 ns each; only replicates with stable secondary structures were used for further analysis. The GROMACS 2023.4 software package was used to model and analyze MD trajectories. Simulations were performed in an explicit solvent in the parmbsc1 force field [46] at T = 300 K under the control of a velocity conversion thermostat [105], with isotropic constant pressure boundary conditions under the control of the Berendsen pressure coupling algorithm [106], and applications of the Ewald particle mesh [107] method for long-range electrostatic interactions (PME). The TIP4P triclinic box [108] of water molecules was added around the DNA to a depth of 25 Å on each side of the solvent. The negative charges of the systems were neutralized by the addition of sodium cations, resulting in an ion concentration of ∼0.15 M. Sodium and chloride ions were added to the systems by substituting water molecules at random positions with a minimum ion spacing of 6 Å.

### 4.3. Cell Cultures

Primary cultures of human glioblastoma cells G01 and BU881 and primary cultures of olfactory neuroepithelial lining cells OES-b were obtained from explants provided by the N.N. Burdenko National Medical Research Center for Neurosurgery (Moscow). This study was approved by the Ethics Committee of Burdenko Neurosurgical Institute, Russian Academy of Medical Sciences (№_12/2020). All subjects gave written informed consent in accordance with the Declaration of Helsinki. The cells were cultured in DMEM/F12 medium (Servicebio, Wuhan, Hubei, China) supplied with 10% FBS (Biowest, Nuaille, France), 2 mM L-glutamic acid, (Paneco, Moscow, Russia), and 1% antibiotic solution (penicillin/streptomycin) (Corning, Corning, NY, USA) at 37 °C and 5% CO_2_. Cells were removed from culture vessels using the Versene solution (Paneco, Moscow, Russia) and 0.25% Trypsin solution (Paneco, Moscow, Russia).

### 4.4. Cell Cultivation with Aptamers

Aptamers were used in in vitro experiments at a concentration of 10 μM. Prior to addition to cell culture, aptamers were treated at 95 °C followed by cooling at room temperature for one hour. Cells were treated with the aptamer for 72 h.

### 4.5. MTS Assay

Changes in cell proliferative activity after exposure to aptamers were assessed by MTS assay. Cells were seeded at 2000 cells per well in 96-well plates (three replicates) in DMEM/F12 culture medium. Incubation was performed at 37 °C with 5% CO_2_ for 72 h. After 72 h, the cells were washed and 100 μL culture medium plus 10 μL MTS reagent (Promega, Madison, WI, USA) was added per well. Cells were incubated at 37 °C with 5% CO_2_ for 2 h. Aptamers were not present in the positive control; the cell medium was used as a blank. Optical density was measured at l = 495 nm using a CLARIOstar Plus tablet analyzer (BMG LABTECH, Ortenberg, Germany).

### 4.6. Transcriptome Analysis

G01 glioblastoma cells were treated with the Gol1 aptamer as described above. For transcriptome analysis, cells were treated with Trizol reagent (Thermo Fisher Scientific, Waltham, MA, USA) according to the protocol. The quality and quantity of total RNA were assessed using a BioAnalyzer and RNA 6000 NanoKit (Agilent, Waldbronn, Germany). The PolyA fraction was then isolated from the total RNA using the Dynabeads^®^ mRNA Purification Kit (Ambion, Austin, Texas, USA; Thermo Fisher Scientific, Waltham, MA, USA) oligoT magnetic beads according to the kit protocol. PolyA RNA libraries were then generated using the NEBNext^®^ RNA UltraII Kit (NEB, Hitchin, UK), quantified using the Qubit dsDNA HS Assay Kit (Thermo Fisher Scientific, Waltham, MA, USA) on a Qbit 2.0 instrument, and fragment length distribution analysis using the Agilent High Sensitivity DNA Kit (Agilent, Waldbronn, Germany). Sequencing was performed on a HiSeq1500 instrument (Illumina, San Diego, CA, USA) with a minimum of 10 million short reads of 50 nucleotides per sample.

Differentially expressed genes were then calculated using the following algorithm:

1. The reference genome assembly GRCh38.p14 (NCBI RefSeq GCF_000001405.40) was indexed, and RNA-seq data were aligned using STAR (v. 2.7.11). Gene expression levels were obtained using HTSeq (v. 2.0.5).
2. Differential expression analysis was performed using DESEQ2 (v. 1.44.0). For further analysis, only genes with adjusted significance level *p* < 0.05 and |log2FoldChange| > 1 were selected.

Gene enrichment analysis using Gene Ontology terms and the Kyoto Encyclopedia of Genes and Genomes (KEGG) pathway database for differentially expressed genes was performed using the ClusterProfiler package (v. 4.12.0) in R.

### 4.7. RT-qPCR

EGFRvIII has a deletion of exons 2 to 7. The primers for its cDNA were selected at the junction of the remaining exons to avoid annealing on the wild-type matrix. One of the primers to the EGFRwt gene was selected in the deletion zone of the EGFRvIII gene to exclude amplification from the mutant gene matrix. The specificity of the primers was tested on genes cloned into plasmids.

**Figure 13:**
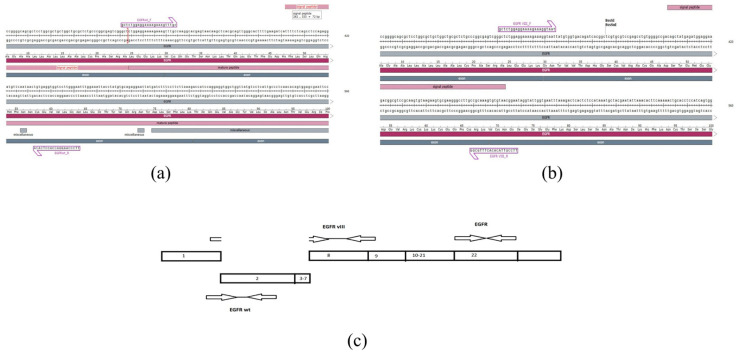
Nucleotide sequence of the primers predicted to match: (**a**) EGFRwt and (**b**) EGFRvIII; (**c**) primer annealing scheme.

Sequence NM_005228.5 was taken as the wild type. This transcription variant encodes the longest isoform. The forward primer is annealed at the junction of exons 1 and 2, and the reverse primer is annealed at the second exon (Figure 13a).

The sequence NM_001346941.2 was taken as EGFRvIII. This variant (EGFRvIII, also known as delta-EGFRwt and de2-7EGFR) has a deletion of six exons in the 5′ coding region compared to variant 1. The forward primer is annealed at the junction of the first and eighth exons, while the reverse primer is annealed at the junction of the ninth and eighth exons (Figure 13b).

The primer annealing scheme is shown in Figure 13c.

To determine the expression level of EGFR and EGFRvIII, total RNA was isolated from human glioblastoma G01 and BU881 cells using the RNAzolⓇ RT reagent (Sigma, Milwaukee, WI, USA) according to the manufacturer’s protocol. MMLV H minus reverse transcriptase (Thermo Fischer, Waltham, MA, USA) and N10 random primer (Eurogen, Eurogen, Russia) were used to obtain cDNA. The obtained cDNA was used for polymerase chain reaction. PCR-RT parameters: Predenaturation—95 °C for 5 min; 40 cycles of amplification: 10 s denaturation at 95 °C; 30 s primer annealing at 60 °C; 30 s extension at 72 °C; final extension—72 °C for 3 min. Analysis was performed using the LightCycler 96 amplifier and LightCycler 96 software version 1.1 (ROCHE, Basel, Switzerland).

Primers for human genes were selected using the Primer Blast program (NCBI). GAPDH (glyceraldehyde-3-phosphate dehydrogenase) and RPL13A (ribosomal protein L13a) were selected as reference genes. The experiment was performed in three replicates.

The primers used in the study are shown in Table 1.

### 4.8. Statistical Analysis

MARS software 3.33 was used to analyze the MTS assay data, and target gene expression levels were measured using the LightCycler^®^ 96 system software 1.1. GraphPad Prism 9 was used for statistical analysis, data were expressed as mean ± standard error of the mean, and the *p* criterion was considered significant when * = *p* < 0.05, ** = *p* < 0.01, *** = *p* < 0.001, and **** = *p* < 0.0001. The significance of the MTS test was evaluated by one-way analysis of variance (ANOVA) followed by the Bonferroni multiple comparison test. RT-qPCR results were evaluated using multiple unpaired *t*-tests to compare means in two cell cultures.

## 5. Conclusions

In summary, our study introduces a novel way to minimize aptamer structure while preserving elements of secondary structure with a reverse folding search of the most optimal sequence. Precise analysis with molecular dynamics simulations may help us to choose the most attractive conformation putative recognition element. We applied this approach to known aptamers to the EGFR and as a result, we suggest a novel DNA aptamer, Gol1, which is a modified shortened version of the previously known U2 aptamer mapped to EGFRvIII. On human glioblastoma culture cells, the Gol1 aptamer demonstrated a more pronounced antiproliferative effect compared to the U2 precursor, with the aptamers having no statistically significant antiproliferative effect on non-tumor cells. Analysis of changes in transcriptomic data of human glioblastoma cell culture after treatment with the aptamer Gol1 revealed profound changes in gene expression; a significant change in gene expression of the key pro-oncogenic signaling pathways mediated by EGFR was shown. Thus, our study provides a framework to further explore the potential use of the Gol1 aptamer in the therapy and diagnosis of glioblastoma.

## Figures and Tables

**Table 1:** Sequence of oligonucleotide primers.

Gene	Forward Primer 5′-3′	Reverse Primer 5′-3′
EGFRwt	GCTCTGGAGGAAAAGAAAGTTTGC	TTCCCAAGGACCACCTCACA
EGFRvIII	GCTCTGGAGGAAAAGAAAGGTAAT	TTCCGTTACACACTTTGCGG

## Data Availability

The data presented in this study are available on request from the corresponding author. The data are not publicly available due to the reason that some of the generated data are not published.

## Supplementary Materials

The following supporting information can be downloaded at https://www.mdpi.com/article/10.3390/ijms26031072/s1.
