# Supplementary material for: In Silico Born Designed Anti-EGFR Aptamer Gol1 Has Anti-Proliferative Potential for Patient Glioblastoma Cells
Source: Int J Mol Sci. 2025 Jan 26;26(3):1072. doi: 10.3390/ijms26031072 (PMC11817825; doi:10.3390/ijms26031072)
Supplement: Supplementary file 1 [file ijms-26-01072-s001.zip › gol1-publ (1).html]

gol1-publ


## Imports and functions¶

In [2]:

```
import RNA
from collections import defaultdict
import pandas as pd
import matplotlib.pyplot as plt
import matplotlib.cm as cm

import varnaapi
from varnaapi import Structure
varnaapi.set_VARNA('/tmp/VARNAv3-93.jar')

import numpy as np

from IPython.display import display, Image, SVG
import ipywidgets as widgets

RNA.cvar.uniq_ML = 1
RNA.cvar.gquad = 1
RNA.cvar.noGU = 0
RNA.params_load_DNA_Mathews2004


min_len = 5
max_len = 35
N = 3
```

In [3]:

```
def drawrna(s,ss,save=None):  
    v = Structure(structure=ss,sequence=s)
    v.show(800)
    if save :
        v.savefig(save)
    #display(svg)
    return True
def draw_ss(sequence):
    ss, mfe = RNA.fold(sequence)
    RNA.svg_rna_plot(sequence,ss,'/tmp/1.svg')
    svg = SVG('/tmp/1.svg', )
    print(mfe)
    #display(svg)
    return svg
def draw_varna(sequence,color="lightblue",save=None):
    ss,mfe = RNA.fold(sequence)
    v = Structure(structure=ss,sequence=sequence)
    v.update(bpStyle='lw', bp='pink', baseNum='black', drawBackbone=False, drawBases=False, fillBases=False, spaceBetweenBases=0.5)
    v.add_highlight_region(1, len(sequence), radius=8, fill=color, outline=color)
    v.show(800)
    if save :
        v.savefig(save)
    
    
def trim_scan(seq,pattern,trimsize=40):
    out = []
    for k in range(trimsize):
        seqs = []
        length = len(seq)
        for i in range(0,k):      
            string = seq[i:length-k]      
            ss, mfe = RNA.fold(string)
            if pattern in ss:
                out.append([length-k,mfe,string])
    out.reverse()
    return pd.DataFrame(out,columns=['i','E','seq'])
```

## Dsiplay known aptamers¶

In [5]:

```
aptamers ='''
U2	ATCCAGAGTGACGCAGCATTTTGACGCTTTATCCTTTTCTTATGGCGGGATAGTTTCGTGGACACGGTGGCTTAGT	3.37±0.98
U8	ATCCAGAGTGACGCAGCATGAATCTTTTCTTTTGGTTTTGATATTTATAGTTGGTGAATGGACACGGTGGCTTAGT	4.35±1.17
U19	ATCCAGAGTGACGCAGCATTTGTATCCTATTTTGTTTATGTAATTGTCGTTGATCATGTGGACACGGTGGCTTAGT	16.78±5.90
U31	ATCCAGAGTGACGCAGCATTTGTTTAATATGTTTTTTAATTCCCCTTGTGGTGTGTTGTGGACACGGTGGCTTAGT	8.10±2.36'''
aptamers_data = []
for l in aptamers.split('\n'):
    if 'U' in l:
        aptamers_data.append(l.split('\t'))

for a in aptamers_data:
    # Compute minimum free energy (MFE) and corresponding structure
    ss, mfe = RNA.fold(a[1])
    # Print output
    print(f"{a[0]}:  \n{a[1]}\n{ss}\n SS scrore: {mfe:2.1f}, EGFR Kd: {a[2]}")
draw_varna(aptamers_data[0][1], save='/tmp/u2.png')
```

```
U2:  
ATCCAGAGTGACGCAGCATTTTGACGCTTTATCCTTTTCTTATGGCGGGATAGTTTCGTGGACACGGTGGCTTAGT
..(((..(((.((((((........)))(((((((..((....)).)))))))....)))..)))..)))......
 SS scrore: -14.1, EGFR Kd: 3.37±0.98
U8:  
ATCCAGAGTGACGCAGCATGAATCTTTTCTTTTGGTTTTGATATTTATAGTTGGTGAATGGACACGGTGGCTTAGT
.....((((.((.((((((((((.((.............)).)))))).))))(((......))).)).))))...
 SS scrore: -9.7, EGFR Kd: 4.35±1.17
U19:  
ATCCAGAGTGACGCAGCATTTGTATCCTATTTTGTTTATGTAATTGTCGTTGATCATGTGGACACGGTGGCTTAGT
.....(((((......))))).((..(((((.(((((((((.((((....)))).))))))))).)))))..))..
 SS scrore: -10.4, EGFR Kd: 16.78±5.90
U31:  
ATCCAGAGTGACGCAGCATTTGTTTAATATGTTTTTTAATTCCCCTTGTGGTGTGTTGTGGACACGGTGGCTTAGT
..(((..(((.((((((((...........(((....)))............))))))))..)))..)))......
 SS scrore: -14.5, EGFR Kd: 8.10±2.36
```

## Dsiplay trimed sequence and structure¶

In [7]:

```
## Trim U2
# Set up pattern and apply function to get trim variants
u2 = aptamers_data[0][1]
ss_pattern = '((((((........)))((('
trim = trim_scan(u2,ss_pattern)
minseq = trim['seq'].values[0]

# Draw distribution of trim variant energy against oligonucleotide length  
plt.rcParams.update({'font.size': 16})
colors = cm.Paired(np.linspace(0, 1, len(trim.index)))
fig,axs1= plt.subplots(1,1, figsize=(10, 7), facecolor='w', edgecolor='k' )
trim.plot.scatter(x='i',y='E', s=200, color=colors,ax=axs1)
axs1.set_xlabel("Trim variant sequence length size, nt")
axs1.set_ylabel("Secondary structure score, au")
plt.savefig('/tmp/optimize-gol1.png', dpi=300)
plt.show()

# Draw minimal possible trim 
print(f'Minimal subsequence of U2 with pattern: {ss_pattern}: \n{minseq}\n Sequence lenght: {len(minseq)}')
ss,mfe= RNA.fold(minseq)
draw_varna(minseq,save='/tmp/minseq.png')
```

```
Minimal subsequence of U2 with pattern: ((((((........)))(((: 
AGTGACGCAGCATTTTGACGCTTTATCCTTTTCTTATGGCGGGATAGTTTCGTGGACAC
 Sequence lenght: 59
```

## Generate ne sequences to secondary structure template¶

In [8]:

```
reverse_data = []
for i in range(1000):
    reverse = RNA.inverse_pf_fold(minseq, ss)
    reverse_data.append(reverse)
```

In [12]:

```
# check  output
print(len(reverse_data))
```

Out[12]:

```
1000
```

## Filter generated¶

In [10]:

```
reverse_pd = pd.DataFrame(reverse_data,columns=['seq','dist'])
ss_pattern = '((((((........)))((('
minseqs = []
energies =[]
for s in  reverse_pd['seq'].values:    
    trim = trim_scan(s.replace('U','T'),ss_pattern)
    minseqs.append(trim['seq'].values[0])
    energies.append(trim['E'].values[0])
reverse_pd['minseq'] = minseqs
reverse_pd['minE'] = energies
```

In [13]:

```
# sort by energy 
sorted_rev = reverse_pd.sort_values('minE')
```

### Save generated seqs¶

In [22]:

```
# check  repeates
print('Uniqe count:', sorted_rev['minseq'][:].nunique())
pd.DataFrame(sorted_rev['minseq'][:].unique()).to_csv('reverse_out.csv')
```

```
Uniqe count: 403
```

## Vizualsation 2D generations¶

In [19]:

```
# Draw top 
seen = []
for i in range(10):
    seq = sorted_rev['minseq'].values[i]
    if seq in seen:
        pass
    else:
        display(draw_ss(seq))
        seen.append(seq)
```

```
-26.100000381469727
```

A
G
C
C
G
G
C
G
A
A
A
A
A
A
A
G
C
C
G
C
C
C
G
G
C
A
A
G
C
T
A
G
T
G
C
G
G
C
C
G
G
G
C
A
A
A
A
G
G
C

```
-26.100000381469727
```

A
G
C
C
G
G
C
G
A
A
A
A
A
A
A
G
C
C
G
C
C
C
G
G
C
A
A
G
C
T
T
G
T
G
C
G
G
C
C
G
G
G
C
A
A
A
A
G
G
C

```
-26.100000381469727
```

A
G
G
C
G
C
C
G
A
A
A
A
A
A
A
G
G
C
G
G
G
G
C
G
C
A
A
G
C
T
A
G
T
G
C
G
G
C
G
C
C
C
C
A
A
A
A
G
C
C

```
-26.100000381469727
```

A
G
C
C
G
G
C
G
A
A
A
A
A
A
A
G
C
C
G
C
C
G
G
G
C
A
A
G
C
T
A
G
T
G
C
G
G
C
C
C
G
G
C
A
A
A
A
G
G
C

```
-26.100000381469727
```

A
G
G
C
G
C
C
G
A
A
A
A
A
A
A
G
G
C
G
G
C
G
G
G
C
A
A
G
C
T
A
G
T
G
C
G
G
C
C
C
G
C
C
A
A
A
A
G
C
C

```
-26.100000381469727
```

A
G
C
C
G
G
C
G
A
A
A
A
A
A
A
G
C
C
G
C
C
C
G
G
C
A
G
G
C
T
A
A
T
G
C
A
G
C
C
G
G
G
C
A
A
A
A
G
G
C

In [20]:

```
gol1='GCCGGCATTTTGACGCCGCCCCGGCTGCTTATGCTCCGGGGCATATGGC'
#draw_varna(gol1)
v = Structure(structure=RNA.fold(gol1)[0],sequence=gol1,)
v.update(background='white',drawTertiary=[10,29],algorithm = 'radiate', 
         autoHelices=True, autoInternalLoops=True, autoTerminalLoops=True,resolution=10)
v.dump_param('/tmp/varnaparm')
v.savefig('/tmp/varna.png')
v.show(800)
Image('/tmp/varna.png')
```

```
autoInternalLoops is not a valid parameter name
A valid argument is one of autoHelices, autoInteriorLoops, autoTerminalLoops, drawBackbone, drawBases, drawNC, drawTertiary, fillBases, flat, backbone, background, baseInner, baseName, baseNum, baseOutline, bp, gapsColor, nsBasesColor, border, bpIncrement, periodNum, resolution, rotation, spaceBetweenBases, zoom, algorithm, bpStyle
```

Out[20]:

## Visualization MD¶

In [7]:

```
params = {"ytick.color" : "k",
          "xtick.color" : "k",
          "axes.labelcolor" : "k",
          "axes.edgecolor" : "k",
          "axes.titlecolor" : "k",
          }
plt.rcParams.update(params)
plt.rcParams.update({'font.size': 16})

fig, axs = plt.subplots(1,3, figsize=(15, 5), facecolor='w', edgecolor='k',
                                    sharex=False,sharey=True,gridspec_kw={'hspace': 0.3, 'wspace': 0.1})
for i,s in enumerate(['aaaa','auau','uuuu']):
    data = np.loadtxt(f'/mnt/storage/golovin/glio-apto/gol1/{s}/2/rmsd-{s}.xvg')
    axs[i].plot(data[:,0]/1000,data[:,1])
    axs[i].set_ylabel('RMSD , nm',fontdict={'size':16})
    axs[i].set_xlabel('time, ns',fontdict={'size':16})
    axs[i].set_title(s.replace('u','t'))
plt.savefig('/tmp/gol1-md.png',dpi=300)
```

In [13]:

```
import pandas as pd
import os
os.chdir('/home/domain/data/golovin/glio-apto/generate-gols')
```

In [26]:

```
data = pd.read_csv('seqs',sep='\t', names=['seq', 'score',])
data['seq'] =  data['seq'].str.replace('U','T')
```

In [31]:

```
d2 = data.sort_values(by='score')
d3= d2[ (d2['score'] < 0.6)  | (d2['score'] >0.8)]
```

In [33]:

```
d3.to_csv('out.csv')
```

In [35]:

```
d3
```

Out[35]:

|  | seq | score |
| --- | --- | --- |
| 90 | GGCGCCGAAAAAAAGGCGGGGGGCTTGCCCATGTTGCCCCCCAAAGGCC | 0.50 |
| 21 | GCCGCGTTCTTTTTCGCGCCCGGCAATCCAACGACGCCGGGCAAATGGC | 0.51 |
| 19 | GCCGGCTTTTTTTTGCCGCCGGGCTTGCCCATGTTGCCCGGCATATGGC | 0.51 |
| 173 | GCCGGCTTTTTTTTGCCGCCCCGCAATCCAACGACGCGGGGCATATGGC | 0.51 |
| 126 | GGCGCCGAAAAAAAGGCGGGCGGCGATCCACTGAAGCCGCCCAAAGGCC | 0.51 |
| ... | ... | ... |
| 189 | GCCGCGGAAAAAAACGCGCCCGGTGTTAGTCATGTGCCGGGCAAATGGC | 0.99 |
| 66 | GGCGGATTTTACTTTCCGGGTCTGTGTAAGTTTGGTAGACCCTATGGCC | 1.04 |
| 140 | GCCGCGGAAAAAAACGCGCCCGGCGTGGAGTTCTGGCCGGGCAAATGGC | 1.11 |
| 165 | GCCGCGTTCTTTTTCGCGCCCGGCGTGGAGTTCTGGCCGGGCAAATGGC | 1.11 |
| 149 | GGCGCCGAAAAAAAGGCGGGAGTGGTGATTTGTCGTACTCCCAAAGGCC | 1.14 |

124 rows × 2 columns

In [ ]:

```
! jupyter nbconvert --to html gol1-publ.ipynb
```

In [ ]:

```

```
