# Supplementary material for: In Silico Born Designed Anti-EGFR Aptamer Gol1 Has Anti-Proliferative Potential for Patient Glioblastoma Cells
Source: Int J Mol Sci. 2025 Jan 26;26(3):1072. doi: 10.3390/ijms26031072 (PMC11817825; doi:10.3390/ijms26031072)
Supplement: Supplementary file 1 [file ijms-26-01072-s001.zip › Supp (1).pdf]

In silico born anti-EGFR aptamer Gol1 has antiproliferative potential for patient glioblastoma cells.

Andrey Golovin, Fatima Dzarieva, Ksenia Rubetskaya, Dzhirgala Shamadykova, Dmitry Usachev, Galina Pavlova and Alexey Kopylov

Supplementary Materials

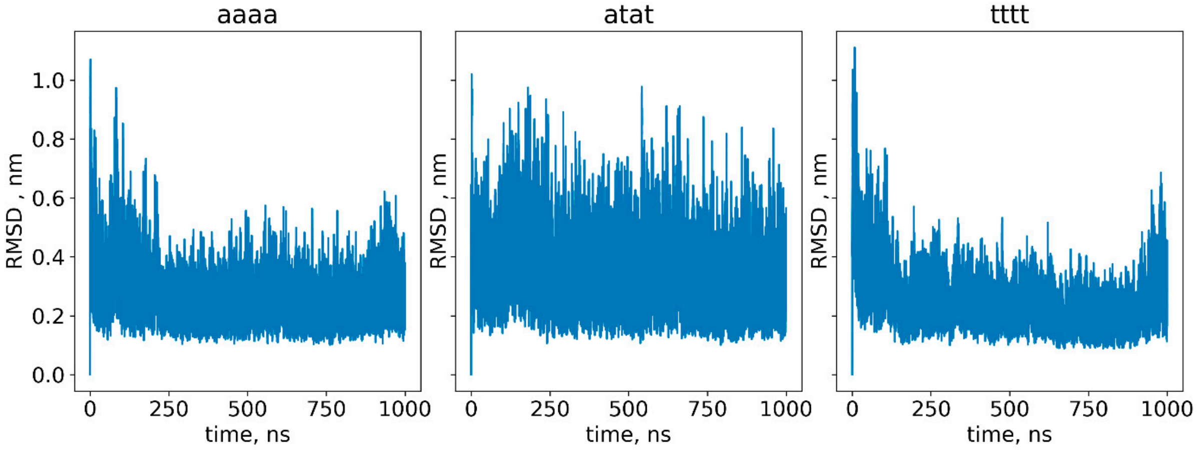

(**a**) (**b**) (**c**)

Figure 1S. RMSD (Root Mean Square Deviation of all DNA atom positions after structure fit) of the selected replica in the MD simulation for (**a**) AAAA content in the junction, (**b**) ATAT content in the junction and (**c**) TTTT content in the junction.

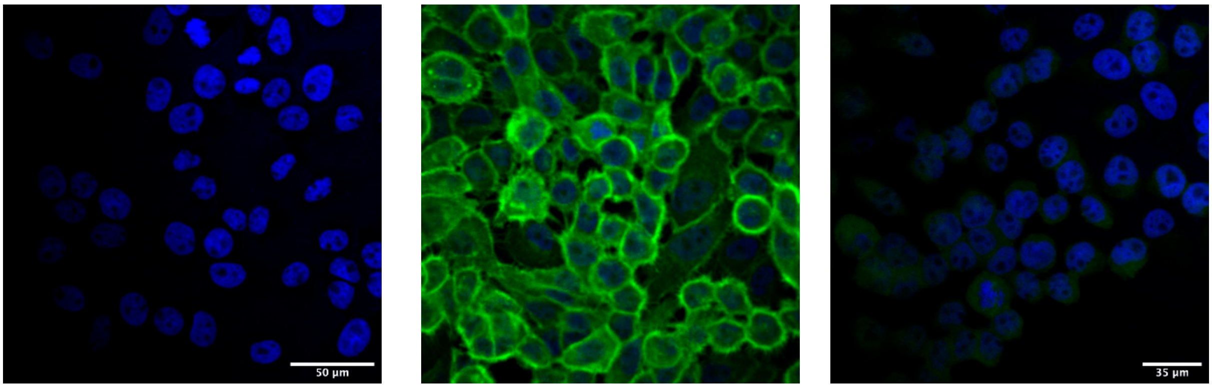

(**a**) (**b**) (**c**)

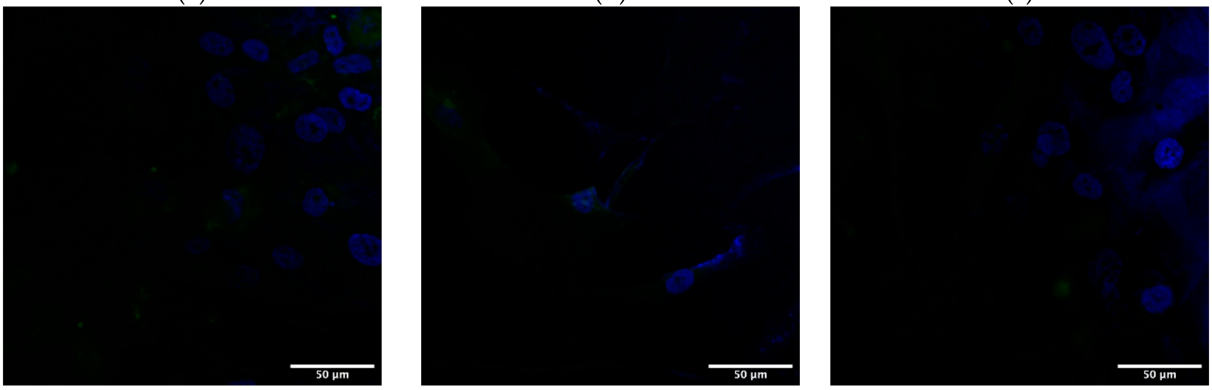

(**d**) (**e**) (**f**)

Figure 2S. Immunocytochemical staining of A431 cells (**a**) nuclei without antibody, (**b**) antibody against EGFR, (**c**) antibody against EGFRvIII and OES-b culture cells (**d**) nuclei without antibody, (**e**) antibody against EGFR, (**f**) antibody against EGFRvIII. Cell nuclei were stained with Hoechst 33342 (blue).

As a positive control to compare the level of EGFR expression, cells from the epidermoid carcinoma cell line A431, which is characterized by a high level of receptor expression, were stained.
